# Sticking around: Cell adhesion patterning for energy minimization and substrate mechanosensing

**DOI:** 10.1016/j.bpj.2022.03.017

**Published:** 2022-03-16

**Authors:** Josephine Solowiej-Wedderburn, Carina M. Dunlop

**Affiliations:** 1 Department of Mathematics, University of Surrey, Guildford, UK; 2 Centre for Mathematical and Computational Biology, University of Surrey, Guildford, UK

## Abstract

Tissue stiffness (Young’s modulus) is a key control parameter in cell behavior and bioengineered gels where defined mechanical properties have become an essential part of the toolkit for interrogating mechanotransduction. Here, we show using a mechanical cell model that the effective substrate stiffness experienced by a cell depends, not just on the engineered mechanical properties of the substrate but critically also on the particular arrangement of adhesions between cell and substrate. In particular, we find that cells with different adhesion patterns can experience two different gel stiffnesses as equivalent and will generate the same mean cell deformations. In considering small patches of adhesion, which mimic focal adhesion complexes, we show how the experimentally observed focal adhesion growth and elongation on stiff substrates can be explained by energy considerations. Relatedly, energy arguments also provide a reason why nascent adhesions do not establish into focal adhesions on soft substrates, as has been commonly observed. Fewer and larger adhesions are predicted to be preferred over more and smaller, an effect enhanced by random spot placing with the simulations predicting qualitatively realistic cell shapes in this case.

## Significance

Experimentally, cell mechanotransduction and stiffness sensing are commonly investigated using engineered gel substrates with defined stiffness. Here, we show, using a theoretical model incorporating active cellular contractility, that cell stiffness sensing depends critically not just on the stiffness of the gel but also on the spatial patterning of adhesion sites. This suggests a need to control cell adhesion as well as gel stiffness in cell biophysics experiments. By considering the model predictions of substrate strain energy we show that it is energetically favorable for focal adhesions to grow and elongate on stiff substrates but that this is not the case on soft substrates. This matches experimental observations of focal adhesion dynamics and provides an explanation for this effect.

## Introduction

It is becoming increasingly apparent that mechanical cues play an important role in controlling cellular behavior affecting, for example, the growth, differentiation, and ultimate fate of cells (1, 2, 3, 4, 5). This ability of a cell to respond to a mechanical stimulus is known as mechanotransduction. Experimental investigations of mechanotransduction commonly focus on stiffness as a single control parameter. This has stimulated activity in developing biomaterials with defined stiffness, ligand density, and functionalization (6, 7, 8). As well as the changes in behaviors mentioned, several cellular structural changes have been identified that occur in response to changes to substrate stiffness. Cell shape is observed to be altered on soft versus stiff substrates, with cells adopting smaller, rounder shapes on softer substrates and appearing larger and more angular on stiffer substrates (9, 10, 11, 12, 13). The distribution of adhesion sites and their size has equally been found to be dependent on gel stiffness. For example, finding that the size of focal adhesions (FAs) increase on stiffer substrates (10,12,14,15), whereas on soft substrates nascent adhesions do not stabilize into FAs (11,16). The effect of cell shape and geometric constraints on cell behaviors have further been investigated using micropatterning techniques (17). In these studies, surface functionalization is used to constrain cell adhesion to predefined regions (18). A significant set of studies have focused on areas of complete adhesion in specific geometries (e.g., circular, triangular, square) (19,20), these have demonstrated that adhered area and shape can control a range of cellular behaviors, including proliferation and signaling (5,18,21). Further studies have used micropatterning to uncouple the effects of adhesion size and cell spreading (22,23).

As microscopy and biophysical tools advance, we are beginning to understand the structure of cells and their ability to generate forces (24). Central to this and much studied is the integrin binding of cells to the extracellular matrix. A particular focus of mechanobiology has been the FA complexes, which generate small patches of strong attachment between the cell and the gel on sufficiently stiff substrates (15). (For details of the structure of the cytoskeleton and FA dynamics, see reviews (16,25, 26, 27).) Cells generate active contractile forces while attached to the underlying substrate (10,28,29). These forces are generated by myosin motors and are transmitted to the extracellular matrix through the adhesions (30,31). The substrate resistance experienced consequently allows the cell to mechanically sense its environment although the details of the physical mechanism of this signal transduction remains unclear. FAs have received most attention as mechanosensors (32, 33, 34), although there is an increasing awareness of the need to account for mechanosensing across the cell including at the nuclear envelope (35,36).

Several theoretical models have been developed to gain an insight into cellular force generation and its effects (37, 38, 39, 40, 41, 42, 43). Largely, these treat the main body of a cell as an elastic solid which is being acted upon by an active component; this is coupled to a substrate which offers further resistance to the force. The way in which the active cellular contractility is represented broadly falls into two categories: simulations of cytoskeletal dynamics and active continuum theories. Computational simulations of the cytoskeleton tend to focus on the dynamics of subcellular constituents of the contractile mechanism to investigate the cell-scale effects of their collective behavior (e.g., (39, 40, 41)). In the continuum approach, an active contractile term is added to the material constitutive relations and, in this way, either a force balance equation (42,43) or an equivalent energy minimization (44) problem can be constructed for the cell deformations and stress. This is referred to as the active stress approach, with active stresses incorporated into different constitutive relations including linear elastic (42, 43, 44) and viscoelastic materials (37). Such models can also be adapted to incorporate different adhesion dynamics, including FA clustering (45), and to investigate intracellular mechanics (12).

In this work, we adopt a continuum mechanics approach modeling cellular contractility as an active stress, reducing the model to two dimensions under the assumption of plane stress. We focus on the significance of adhesion distribution and patterning on a cell’s overall ability to deform. Two cases are considered. First, we consider the paradigm model that the cell is adhered in a ring around its edge before considering the case that the cell is adhered at several distinct spots which mimic FA complexes. In the case of spot adhesion we vary the distribution, total area, and shape of adhered regions and consider the effects on the mean cellular deformation, relating this to the resistance the cell experiences from the underlying substrate. These results show that the substrate may be experienced as more or less stiff depending on how the cell is adhered. Specifically, we show that a cell with a sparse distribution of adhered regions around its periphery, with large gaps between them, effectively experiences a softer substrate than a cell with more continuously distributed adhesions around its edge. Cell morphology is also observed to change qualitatively agreeing with experimentally observed shape changes when the adhesion points are randomly distributed. Indeed energy calculations show that it is energetically favorable, reducing the work done to the substrate, for the cell to be adhered at points with large variance in interspot spacing. Significantly when considering substrate strain energy we find that it is also energetically favorable for the sites of adhesions to grow and elongate on stiff substrates matching the commonly accepted dynamics of FAs on stiff substrates. We also show that, on soft substrates, growing adhesions is not energetically favorable, which provides an explanation for the observation that, on soft substrates, nascent adhesions do not form into stable FAs.

## Methods

### Theoretical model

We use the continuum mechanics active stress formulation for modeling contractile cells on soft gel substrates (22,42,44,46, 47, 48). Thus the cell stress $\sigma =\sigma^{P}+\sigma^{A}$, where $\sigma^{P}$ is the passive stress generated through the deformation of the cell and $\sigma^{A}$ is an active component of stress generated by the contractile machinery embedded in the cytoskeleton. We assume that the cell has attained a spread area with a much larger radial length $r_{0}$ than thickness, *h*, and so make the plane stress assumption. Furthermore, we assume a linear elastic constitutive equation for passive stress $\sigma_{ij}^{P}=\frac{hE_{c}}{\left(1+\nu \right)}\left(\varepsilon_{ij}+\frac{\nu }{1-\nu }\varepsilon_{kk}\delta_{ij}\right)$, where $\varepsilon_{ij}$ denotes the cellular strain in two dimensions i,j=1,2 and the summation convention is applied to repeated indices so that $\varepsilon_{kk}=\varepsilon_{11}+\varepsilon_{22}$. The cell Young’s modulus and Poisson’s ratio are denoted by $E_{c}$ and ν, respectively. The active contractile pressure is assumed isotropic and so $\sigma^{A}=\frac{hE_{c}}{2\left(1-\nu \right)}P_{0}\delta_{ij}$, with $P_{0}$ representing a target contraction and we assume throughout that $P_{0}$ is constant.

The contraction of the cell is resisted by its attachment to the underlying gel substrate and it is from this force balance that the cell deformations are determined. We assume a linear elastic response in the gel in addition to the cell, noting that the timescale for cell adhesion is faster than the relaxation timescale (44) so that viscoelastic effects may be neglected. However, over longer timescales viscoelasticity could lead to stress relaxation in the gel (49). We model cell adhesion to the underlying substrate through the force balance (with plane stress in the cell layer)

(1) ∇⋅σ−KT(x)u=0,

with T(x)=0 where the cell is not adhered and T(x)=1 where the cell is adhered. In the case of uniform adhesion (T(x)≡1) we recover the force balance considered in (42,48). In this model, the resistance of the substrate is assumed proportional to the deformation with the constant of proportionality *K* depending on substrate stiffness. This is a common first-order approximation for thin gel substrates (e.g., (42,48,50, 51, 52)). A formal justification of this approximation is presented in (47), however, it may be intuitively understood as when the gel layer is thin the attachment to the rigid solid base localizes the deformations near the sites of applied traction generating an approximately linear relationship between stress and deformation as for a spring. In (47,48), *K* is related to the material properties of the gel, with $K\approx E_{S}/2\left(1+\nu_{S}\right)h_{S}$ for a gel with Young’s modulus $E_{S}$, Poisson ratio $\nu_{S}$ and thickness $h_{S}$ (47,48). Note that, in the case of micropillar assays, see (6,53), the resisting stress is localized to each pillar and is proportional to the deformation of the pillar so that K=Nk where *k* is pillar stiffness and *N* the number density of pillars.

**Figure 1:**
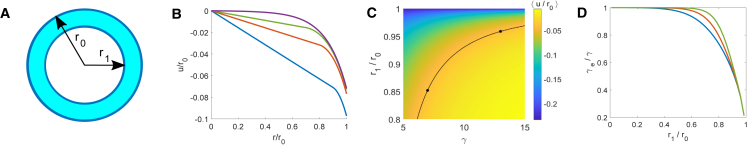
Adhesive area changes the effective substrate stiffness experienced. (*A*) Schematic diagram of a circular cell with an adhesive ring. (*B*) Plot of deformation profile of a cell with $r_{1}/r_{0}=0.9,0.8,0.7,0$ (from bottom to top). $r_{1}/r_{0}=0$ corresponds to complete adhesion (γ=7). (*C*) Heatmap showing how mean cellular deformation varies with ring thickness (parameterized by $r_{1}$) and substrate stiffness (parameterized by γ). Along the contour line (*black*), cells display the same mean deformation. (*D*) Relative effective resistance plotted against internal ring radius ($r_{1}$) on substrates with γ=5,10,15 (from bottom to top). (Here and in all further figures we set $P_{0}=0.7$ and ν=0.45.) To see this figure in color, go online.

We here consider two cases for T(x). The first is that the cell is completely adhered around its edge and the second that the cell is adhered at distinct spots that are spatially distributed around the edge. The first case models micropatterning experiments (e.g., (18,48)) where the geometry of adhesion is tightly controlled and usually restricted to simple shapes, the latter case can be considered to describe FAs. In the paradigm example of a ring of adhesion (see Fig. 1 *A*),

$$T\left(x\right)=\left\{ \begin{array}{cc} 0, & \left|x\right|<r_{1}\\ 1, & \left|x\right|\in \left[r_{1},r_{0}\right]. \end{array}\right.$$

In this case deformations are purely radial so that $u=u\left(r\right)e_{r}$. This has the benefit that the force balance Eq. 1 with a no stress boundary condition and suitable continuity conditions at the internal boundary can be solved analytically to give the solution (full derivation in Supporting material)

$$\frac{u}{P_{0}r_{0}}=\left\{ \begin{array}{cc} \alpha_{0}\frac{r}{r_{0}}, & r\in \left[0,r_{1}\right)\\ \alpha_{1}I_{1}\left(\frac{\gamma r}{r_{0}}\right)+\beta_{1}K_{1}\left(\frac{\gamma r}{r_{0}}\right), & r\in \left[r_{1},r_{0}\right], \end{array}\right.$$

where $I_{1}$ and $K_{1}$ are modified Bessel functions, and

$$\alpha_{0}=\frac{r_{0}}{r_{1}}\left(\alpha_{1}I_{1}\left(\frac{\gamma r_{1}}{r_{0}}\right)+\beta_{1}K_{1}\left(\frac{\gamma r_{1}}{r_{0}}\right)\right),\;\alpha_{1}=-\frac{\left(1+\nu \right)}{2\gamma }\cdot {\left(F\left(\gamma \right)-G\left(\gamma \right)H\left(\frac{\gamma r_{1}}{r_{0}}\right)\right)}^{-1},\;\beta_{1}=H\left(\frac{\gamma r_{1}}{r_{0}}\right)\alpha_{1}.$$

For conciseness we have introduced functions:

$$F\left(z\right)=I_{0}\left(z\right)+\frac{\left(\nu -1\right)}{z}I_{1}\left(z\right),\;G\left(z\right)=K_{0}\left(z\right)-\frac{\left(\nu -1\right)}{z}K_{1}\left(z\right),\;H\left(z\right)=\left(\frac{zI_{0}\left(z\right)-2I_{1}\left(z\right)}{zK_{0}\left(z\right)+2K_{1}\left(z\right)}\right),$$

see Fig. 1 *B*. The contraction parameter $P_{0}$ is seen to linearly scale the deformation altering its magnitude only. There is one nondimensional parameter, $\gamma^{2}=K\left(1-\nu^{2}\right)r_{0}^{2}/hE_{c}$, which quantifies the substrate resistance compared with that of a cell with Young’s modulus $E_{c}$, Poisson ratio ν, diameter $2r_{0}$ and thickness *h*. For a stiffer substrate (*K* larger) γ is greater. As a specific example for a gel of thickness 35 *μ*m with Young’s modulus $E_{S}=70$ kPa and indicative cell parameters $E_{c}=10$ kPa, $r_{0}=30$ *μ*m, h=1 *μ*m, and $\nu =\nu_{S}=0.45$, γ=7 (see Table S1, Supporting material).

**Figure 2:**
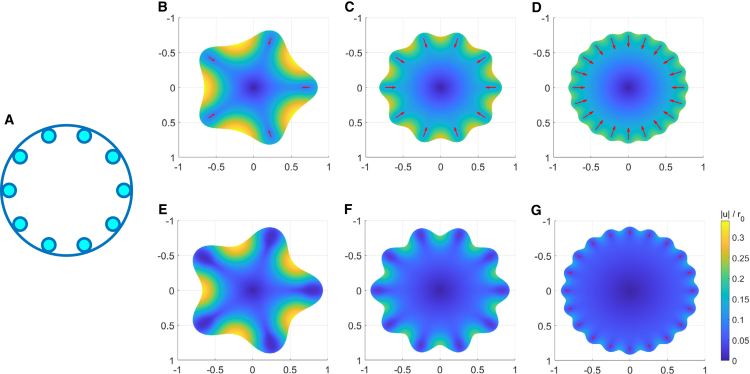
Arrangement of adhesion into localized spots facilitates localized regions of high deformation. (*A*) Schematic diagram of a circular cell with 10 spots evenly distributed around the edge. Heatmaps of the mean deformation on soft substrates with γ=5 (*B–D*) or stiff with γ=11.34 (*E*–*G*) with arrangements of 5 spots ($r_{s}=0.14r_{0}$) (*B* and *E*), 10 spots ($r_{s}=0.1r_{0}$) (*C* and *F*), and 20 spots ($r_{s}=0.07r_{0}$) (*D* and *G*) evenly distributed around the cell edge. Adhered area is maintained at 10% *A*, where *A* is the precontraction cell spread area (i.e., $A=\pi r_{0}^{2}$). Red arrows show the deformation of the midpoint of each spot. To see this figure in color, go online.

To model localized spots of adhesion and in particular FAs, we take T(x)=1 only in small circular or elliptical regions in the cell (e.g., Fig. 2 *A*). In this case, an analytical solution cannot be obtained and numerical solutions are obtained using finite element methods, see as an example Fig. 2. However, in the case of spots it can be seen from the linearity of Eq. 1 that again γ is the key control parameter quantifying the substrate resistance, with $P_{0}$ giving a scale for the magnitude of deformation.

### Numerical methods

All numerical solutions were obtained using finite element methods implemented within MATLAB (specifically using PDE Toolbox R2018a) for the elliptic PDE Eq. 1 with von Neumann boundary conditions (for further details of the implementation see Supporting material). The cell geometry was specified and a triangular mesh generated. To calculate the integral for the mean cellular deformation and energy integrals from the numerically computed data the Gaussian quadrature of degree 1 was used to approximate the solution on each triangle of the mesh. After defining the geometry the problem can be completely parametrized by γ, $P_{0}$, and ν for a general adhesion geometry. We vary γ and take ν=0.45 throughout.

The contractility parameter $P_{0}$ only scales the deformation *u*; however, we specifically take $P_{0}=0.7$ throughout. Although $P_{0}$ may be expected to vary between cell experiments, that this is an appropriate scale can be seen by comparison with, e.g., (54), where $P_{0}=0.7$ generates the same contractile moment as reported for a gel substrate of thickness 30 *μ*m and $E_{s}=4$ kPa (γ=3). The net contractile moment is given by $M_{ij}=\frac{1}{2}\int d^{2}r\left[x_{i}T_{j}\left(r\right)+x_{j}T_{i}\left(r\right)\right]$ (55), where T(r) is the traction force which may be calculated from the deformations. We set cell parameters as above (see also Supporting material), with $r_{0}=50$ *μ*m (to match an observed deformed cell area of 4100 *μ*m^2^) taking also 100 adhesions, aspect ratio 2, evenly distributed around the cell edge covering 10% cell area. In this case, our model with $P_{0}=0.7$ predicts the average experimentally determined net contractile moment of ∼13 pNm.

For a regular arrangement of spots circles of adhesion were placed at angles 2π/N, where *N* is the number of spots, see Fig. 2 *A* for an example; the spots are placed radially at a position $0.98r_{0}-r_{s}$, where $r_{0}$ is the radius of a circular cell and $r_{s}$ is the spot radius. To simulate nonuniform spot distributions (see Fig. 5) we consider two cases. In both instances we considered 20 spots, each the same size and totaling an adhered area of 10% of the cell spread area. First, we distributed the spots around the edge of a cell (assumed to be circular with radius $r_{0}$), dividing a circle of radius $0.91r_{0}$ into 37 “bins” such that each was large enough for 1 spot with no overlaps. In each of our simulations, 20 bins were randomly sampled, resulting in a distribution of 20 spots around the cell edge (see Fig. 5, *C* and *E*). Second, we considered spots distributed in an annular region located near the cell edge (see Fig. 5, *D* and *F*). For direct comparison of the effect of variation in the radial position of the spots as opposed to located at the edge, we considered the same angular distributions of spots as in the edge simulations. The radial position of the center of each spot was randomly sampled from a uniform distribution between 0.6 $r_{0}$ and 0.91 $r_{0}$. The distance between adjacent spots was tested to ensure there was no overlap. In cases where the simulations did result in distributions with overlapping spots, the radial position of spot to the “left” was resampled until there was no overlap.

## Results

### Adhesive area and arrangement changes the effective substrate stiffness experienced

Considering first micropatterning experiments (e.g., (18,48)) and specifically the paradigm example of cells adhered uniformly at their outer edge, we observe in Fig. 1 *B* that the cell’s ability to deform is affected by the width of its adhesive ring. Specifically that thicker rings exhibit reduced deformations. To better quantify the potential effect of adhered area on mechanosensing it is necessary to define a measurable quantity for comparison. One such measure adopted, which is relatively easy to interpret, is the mean deformation over the cell area (56). Where the mean deformation is lower the apparent substrate stiffness by this measure would be higher, whereas a larger mean deformation would correspond to a softer substrate. For the adhered ring the mean cellular deformation (scaled by cell radius) can be explicitly calculated, see the Supporting material.

Fig. 1*C* shows how the mean cellular deformation varies with the adhesive ring width and substrate resistance parameter γ. Cells with the thinnest adhered rings and on the least resistant substrates display the greatest mean cellular deformation. Also plotted in Fig. 1 *C* is an illustrative contour along which the mean deformation is constant ($\left\langle u/r_{0}\right\rangle =-0.04$). Specifically, it can thus be seen that a cell with $r_{1}/r_{0}\approx 0.85$ on a substrate γ=7 experiences the same mean deformation as a cell with $r_{1}/r_{0}\approx 0.96$ on a substrate γ=13 and thus potentially senses both as equally stiff.

A different way of conceptualizing this difference is that a cell with ring of adhesion thickness $r_{1}/r_{0}=0.85$ on a substrate engineered at stiffness γ=7 experiences this substrate as if it were of stiffness γ≈4.6 (choosing as reference the state of full adhesion). This observation informs our definition of an *effective substrate stiffness* $\gamma_{e}$ for the adhesive pattern, which is the γ on the contour of constant mean deformation corresponding to complete adhesion. $\gamma_{e}$ may be calculated by solving $\left\langle u\left(\gamma \right)\right\rangle =\left\langle u_{CD}\left(\gamma_{e}\right)\right\rangle$, where $u_{CD}$ is the solution for a completely adhered disc. This solution is obtained numerically with a Newton-Raphson iterative scheme and plotted in Fig. 1 *D*. We see that throughout $\gamma_{e}<\gamma$, so that the resistance experienced by the partially adhered cell is less than that for a completely adhered cell. Specifically, cells with thinner rings (with $r_{1}/r_{0}$ near 1) sense an effectively softer substrate as they experience less resistance; however, this effect becomes less pronounced for wider adhesive rings. For example, when $r_{1}\approx 0.66r_{0}$, $\gamma_{e}/\gamma$ is already close to 1 ($\gamma_{e}/\gamma =0.9$ on γ=5), showing that the cell is sensing almost the same resistance as it would when completely adhered. This effect is enhanced on stiffer substrates, for instance, when γ=15, $\gamma_{e}/\gamma =0.9$ is obtained at $r_{1}\approx 0.78r_{0}$ (see Fig. S1).

Other measures that may correlate with mechanosensing demonstrate very similar heatmaps to Fig. 1 *C*, with reduced adhesion resulting in a lower effective stiffness with a similar dependency on substrate stiffness. This is to be expected given the close relationship between deformation, strain, and energy arguments. See, for example, Fig. S2, where the effect of changing adhesion on maximal cellular deformation, mean cellular strain, and maximal cellular strain (46) are considered with energy arguments considered in a later section.

To consider the effect of breaking up cellular adhesion into small spots of adhesion (mimicking the FAs), we introduce a distribution of circular spots placed around the cell periphery. We isolate the effects of adhesion distribution, varying the number of spots but maintaining a constant adhered area, this increases the between spot gaps. The outputs of simulations with 20, 10, and 5 spots on soft and stiff substrates are shown in Fig. 2, *B*–*G*. The corresponding mean cellular deformations and effective resistance parameters are given in Table 1. It is clear that reducing the number of adhesions increases the gap size between adhered regions, thus facilitating localized areas of high deformation. On the stiffer substrate the cellular deformations are, as expected, smaller. Where the gaps are larger, however, the effect of substrate stiffness is reduced. Considering, for example, the maximal deformation with 20 spots we find a 40% decrease in the maximum deformation from γ=5 to γ=11.34, while with 5 spots the decrease in maximum deformation is only 5% with the same increase in substrate stiffness.

**Table 1:** Arrangement of adhesion into localized spots reduces the effective stiffness of the substrate

	γ=5		γ=11.34	
	$\left\langle u/r_{0}\right\rangle$	$\gamma_{e}$	$\left\langle u/r_{0}\right\rangle$	$\gamma_{e}$
20 spots	0.133	0.37γ=1.84	0.055	0.33γ=3.75
10 spots	0.144	0.33γ=1.67	0.084	0.25γ=2.82
5 spots	0.166	0.27γ=1.35	0.133	0.16γ=1.84

Mean cellular deformation and corresponding effective resistance parameter for different spot distributions parameters as for Fig. 2.

In Table 1, we present the mean cellular deformations corresponding to the simulations in Fig. 2. We see that, on substrate γ=5, halving the number of spots from 20 to 10 increases the mean deformation by 8.3%; thus, demonstrating that decreasing the number of adhered spots results in an increased mean cellular deformation so that by decreasing the number of adhered regions a cell effectively senses a softer substrate. This effect is enhanced on stiffer substrates, for example, when γ=11.34 halving the number of spots from 20 to 10 increases the mean deformation by 52.7%. Furthermore, a cell with 20 spots on substrate γ=5 may effectively sense the same resistance ($\gamma_{e}=1.84$) as with 5 spots on substrate γ=11.34. We reiterate that the adhered area is the same in each case and here it is the rearranging of the sites of cellular adhesion that enables a cell to experience this softer environment.

### Increasing spot size increases apparent substrate stiffness: An effect which may be compensated for by the elongation of spots into elliptical patches

**Figure 3:**
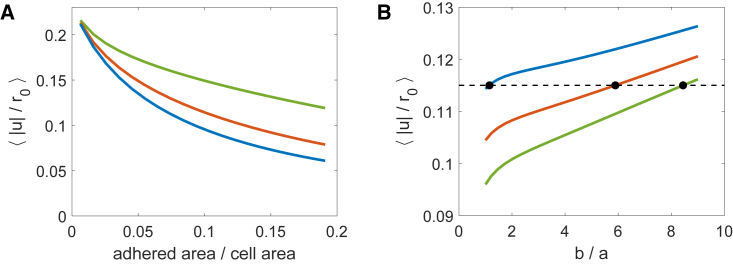
Increasing spot size increases apparent substrate stiffness: an effect which may be compensated for by the elongation of spots into elliptical patches. (*A*) Mean deformation plotted against adhered area for evenly distributed adhesive spots, with number of spots 5, 10, and 20 (from top to bottom). (*B*) Mean deformation plotted against spot aspect ratio for 10 evenly distributed spots with adhered area $A_{\text{ad}}=10\text{\%},12\text{\%},14\text{\%}$ (from top to bottom). Increasing the aspect ratio b/a corresponds to an elongation toward the center of the cell. The black dotted line indicates $\left\langle |u|/r_{0}\right\rangle =0.115$. (In (*A*) and (*B*) γ=7.) To see this figure in color, go online.

Increasing individual spot size while keeping the number of adhesions fixed has the natural effect of increasing the adhered area. As such we find that the mean cellular deformation decreases with the increased resistance from a greater adhered area Fig. 3 *A*. Correspondingly, we suggest that the cell effectively experiences a stiffer substrate with an increase in adhered area, in analogy to the results for a cell with an adhesive ring. However, this effect can in part be compensated for if the increase in area is not uniform but is generated through an elongation of the spot. In Fig. 3 *B*, we show how elliptical spots affect the mean deformation. Increasing the aspect ratio of the spots (for a given spot size), we find that elongating the spots inward may result in an increase in the mean cellular deformation (Fig. 3 *B*), potentially compensating for the reduction the increased area has imposed. For example, in Fig. 3 *B* for a cell with 10 spots we see that increasing the adhered area from 10 to 12% without altering the spot shape would decrease the mean deformation substantially; however, by increasing the spot aspect ratio from b/a=1.15 to b/a=5.89 the mean deformation can be kept constant. The mean cellular deformation can similarly be conserved when increasing the adhered area to 14% with a spot aspect ratio of b/a=8.44.

Experimentally it is observed that FAs grow as the applied force increases, and that they additionally tend to elongate in the direction of applied forces (e.g., (15,16,34,57)). Our results suggest that such an elongation may be being used to at least partially compensate for the effect of adhesion growth.

### FA growth reduces strain energy on stiff substrates but not on soft substrates; Elongated adhesion spots are energetically favorable

Considering now the strain energy *W*, of the system, this can be expressed as $W=W_{CA}+W_{CP}+W_{S}$, where $W_{CA}$ is the work done by the active contractile network of the cell, $W_{CP}$ the strain energy in the passive cell components, and $W_{S}$ the substrate strain energy. As this is a closed system, we expect no net loss or gain of energy and so W=0. The active work done thus causes both the deformation of the cell and surrounding substrate. As $W_{CA}=\frac{1}{2}\int_{A}\sigma_{ij}^{A}\varepsilon_{ij}\;dA,$ for constant cellular contractility the active work by the cell is directly proportional to its mean strain. Thus, the behavior of $W_{CA}$ is qualitatively very similar to the mean cellular deformation both for ring adhesion and adhesive spots as discussed above, see Fig. S3. We focus here on the substrate strain energy $W_{S}=\frac{1}{2}\int_{A}KT\left(x\right)u\cdot u\;dA$, which is often experimentally used to quantify the mechanical activity of a cell and its contractile strength (55,58,59). Indeed $W_{S}$ has recently been identified as an important metric to describe the entire output work done across different cell types, morphologies, and substrates (60).

**Figure 4:**
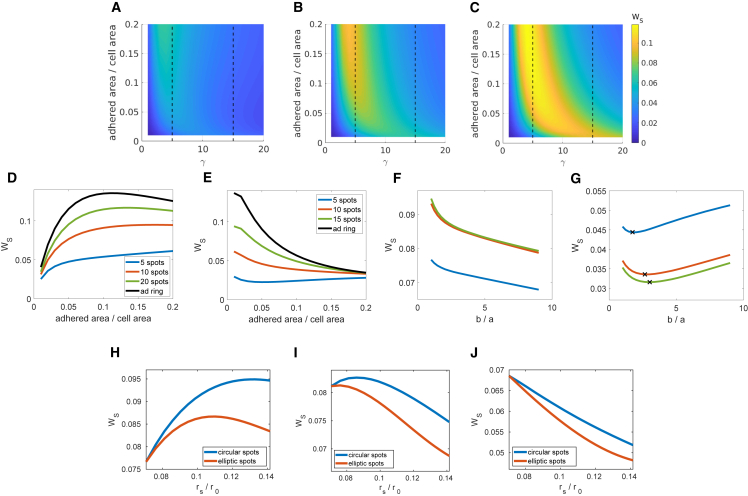
Focal adhesion growth reduces strain energy on stiff substrates but not on soft with spot elongation optimal. Heatmaps showing substrate strain energy against γ and the proportion of adhered area with 5 (*A*), 10 (*B*), and 20 (*C*) adhered spots evenly distributed around the cell edge. $W_{S}$ against adhered area for γ=5 (*D*) and γ=15 (*E*), as indicated by the dotted lines in (*A*–*C*), for 5, 10, and 20 spots. $W_{S}$ for an adhered ring plotted for comparison. $W_{S}$ against spot aspect ratio (b/a) for γ=5 (*F*) and γ=15 (*G*) for 10 spots at adhesion 5, 10, and 15% in blue, orange, and green, respectively. $W_{S}$ for an even distribution of 10 spots on substrates with γ=5 (*H*), γ=7 (*I*), and γ=10 (*J*). The blue line indicates circular spots of spot radius $r_{s}$. The orange line corresponds to elliptical spots with a fixed width but increasing length so that the aspect ratio increases as adhered area increases; here $W_{S}$ is plotted against the equivalent radius of circular spots. (Substrate strain energy is normalized by $hE_{c}r_{0}^{2}/\left(1-\nu^{2}\right)$.) To see this figure in color, go online.

In Fig. 4, *A*–*E* we consider how the adhered area affects $W_{S}$, in particular focusing on the case where spot radius is increased. (For the case of an increasingly wide ring of adhesion see Fig. S4.) In Fig. 4 *D* and *E* we consider the particular cases of a cell adhered to a soft substrate (γ=5) and a stiff substrate (γ=15). For comparison we plot the analytical solution for a completely adhered ring of the same area. We observe that, as expected, the work done to the substrate by cells with adhered spots tends toward the continuous solution of an adhesive ring as the spot distribution becomes more dense. Similarly, when there are fewer adhered regions the cell does less work to the substrate. We observe that on soft substrates the substrate strain energy increases as the adhered area increases (spot radius increases) making this energetically unfavorable, although there is a turning point in this behavior. By contrast, on the stiff substrate it is energetically favorable to increase adhesion size in all of the spot distributions considered. This can be explained as, on stiff substrates, increasing adhesion reduces the potential cell deformation and substrate deformation as the cell is now fixed in place due to the rigidity of the substrate. However, in the case of 5 spots we see a turning point in this behavior, beyond which $W_{S}$ increases as adhesions continue to grow.

Finally, we consider the effects of an elongating spot on the substrate strain energy (shown in Fig. 4, *F* and *G*). In Fig. 4 *F*, we see that on a soft substrate increasing the aspect ratio from circular to elongated elliptic spots (aligned toward the cell center) decreases the work done to the substrate as the cell is more able to deform in the gaps between adhesions. Furthermore, our investigations suggest that on stiffer substrates there is an optimal spot aspect ratio for these adhesions depending on spot size. In the case of an adhered area ranging from 5 to 15% and γ=15 this is approximately two to three times as long as they are wide (Fig. 4 *G*). In Fig. 4, *H*–*J* we consider the effects of increasing the adhered area. Comparing the effect of increasing the radius of circular spots with maintaining a fixed width and accommodating the extra area by elongating the spots toward the center of the cell, we see that elliptic spots result in a lower $W_{S}$. Combined, this suggests the elongation of adhesions is energetically favorable.

### Random placement of adhesion sites can generate apparently softer substrates compared with uniform placement and is energetically favorable

**Figure 5:**
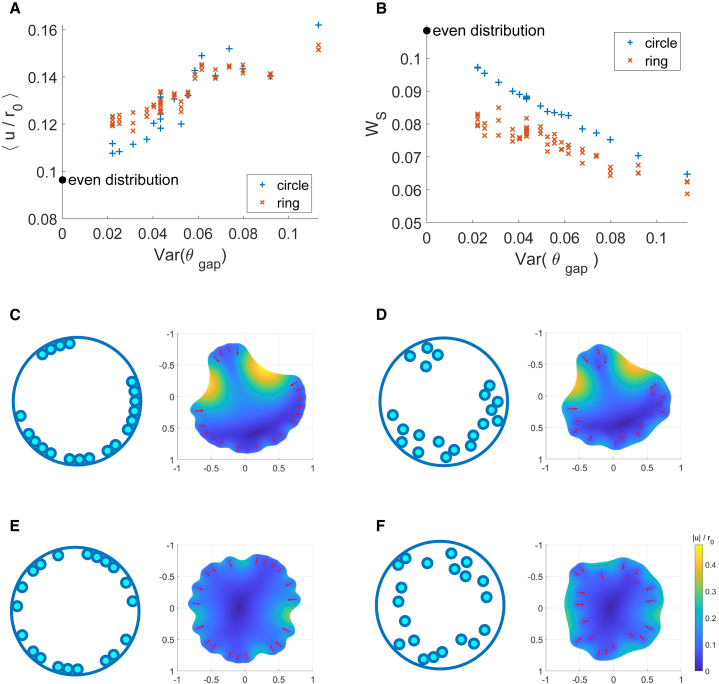
Random placement of adhesion sites can generate apparently softer substrates compared with uniform placement and is energetically favorable. (*A*) Mean cellular deformation and (*B*) substrate strain energy plotted against the variance in angular gap size for spots restricted to the edge (*blue pluses*) and in an annular region ($0.6r_{0}<r<r_{0}$) (*orange crosses*). Results for an even distribution of spots are included for comparison at $\text{Var}\left(\theta_{g}\right)=0$. (In each simulation there are 20 circular spots, covering 10% of the cell spread area, for the ring the same angular spot placements are chosen but the radial position is varied; γ=7.) Examples of the cell deformation observed in the above simulations. (*C*) corresponds to point in (*A*) with largest mean deformation; (*D*) is a corresponding ring distribution; (*E*) corresponds to the point in (*A*) with the least mean deformation; and (*F*) is a corresponding ring distribution. To see this figure in color, go online.

To investigate the effect of more realistic distributions of adhesion in which spots are distributed nonuniformly we considered two cases. In the first, spots are distributed at the cell edge but at a random angle (e.g., Fig. 5, *C* and *E*). In the second, spots are distributed randomly within an annular region located near the edge of the cell (see, e.g., Fig. 5, *D* and *F* and Methods for details of implementation). In all cases, adhesion is considered to localize at the cell edge as is experimentally observed (15,54). With cell adhesion arranged in this manner we observe that the simulated cell shapes (with both annular and ring adhesions patterns) are qualitatively very similar to those observed experimentally (e.g., (8,61)).

In Fig. 5, *C* and *E*, we show the deformation of cells with spot distributions with the greatest and least variance in angles $\left(\theta_{g}\right)$ between adjacent spots from the multiple simulations run for each arrangement. Where $\text{Var}\left(\theta_{g}\right)$ is larger we observe few larger clusters and the existence of larger gaps between clusters, this results in greater mean cellular deformation (see Fig. 5 *A*). In Fig. 5 *B* we see that the substrate strain energy is less where $\text{Var}\left(\theta_{g}\right)$ is larger. This corresponds to the result we found in Fig. 4, *D* and *E*.

To compare the two arrangements (adhesions at the cell edge as compared to within a constrained ring) spot distributions within the ring were chosen to have the same angular distribution (with different radial positions) as the circle distributions in Fig. 5, *A* and *B*. First we see that the mean cellular deformation does not have a clear separation between arrangements of adhesions at the cell edge or within a constrained ring (Fig. 5 *A*). Although, in this particular example we see an increase in mean cellular deformation in 70% of the simulations when spots were moved inward within a constrained ring relative to their corresponding (with the same angular distribution of spots) spot distribution at the cell periphery. There is a relatively even distribution of spots around the cell in the majority of these cases (with Var $\left(\theta_{g}\right)$ ranging between 0.02 and 0.056 for 90% of the ring simulations resulting in a higher mean cellular deformation than their corresponding peripheral simulation). Fig. 5, *E* and *F* illustrate the corresponding cellular deformations. The cell with a distribution of spots around its periphery in Fig. 5 *E* has a lower mean deformation than that of the cell depicted in Fig. 5 *F* with the same angular distribution of spots but some more inwardly located. Conversely, in Fig. 5, *C* and *D* we see an example where the mean cellular deformation is greater in the case where spots are located at the periphery of the cell (Fig. 5 *C*). We explain this higher mean cellular deformation by the regions of particularly high deformation localized to the large gaps (reflected in the high value of Var $\left(\theta_{g}\right)$, recall Fig. 5, *C* and *D* depict the distributions with highest Var $\left(\theta_{g}\right)$ across the simulations presented here) between regions of adhesion in this distribution of spots.

Interestingly, we see a clear separation in the effects of the two arrangements on $W_{S}$ when some adhesions are distributed within a ring at the cell edge (Fig. 5 *B*). However, this is not uniformly the case; for example, when considering the same distributions of spots on a stiffer substrate (γ=15) we find that 20% of the ring simulations result in a higher $W_{S}$ than their corresponding circle simulation; however, for the significant majority of simulations $W_{S}$ is reduced for the ring arrangement, see Fig. S5.

## Discussion

In this study, we have shown in fact that the way in which a cell senses its environment depends critically on how the cell is adhered and not just on the mechanical stiffness of the gel. We use a model based on continuum elasticity with an active stress component to capture cellular contractility, a popular modeling framework for describing contractile cells adhered to substrates (42,44,48). We here extended this model to describe spatial patterns of adhesion, considering two particular classes of cell adhesion patterns. In the first the cell is assumed to be adhered in an annular region at the cell edge, while we model the formation of small patches of adhesion, the experimentally observed FAs, by the introduction of spots of adhesion distributed under the cell primarily near the cell edge. In the case of the adhered ring, analytical solutions are possible and presented, whereas the symmetry breaking inherent in the introduction of adhesive spots necessitates solutions by finite elements.

With a uniform ring of adhesion we show that the mean deformation, a measure commonly experimentally measured (56), increases with a decrease in adhered area (Fig. 1). In this way, an entirely different gel stiffness can generate the same mean deformation if the adhesion percentage is carefully tuned. Similar equivalences across different stiffness have been demonstrated across other measures, including maximal cellular deformation, mean cellular strain, and maximal cellular strain (see Fig. S2). This observation leads us to define an effective stiffness for the combined system of the cell adhered to the gel, which quantifies the stiffness experienced by the cell. We demonstrated that the effective stiffness is always less than the true gel stiffness (Fig. 1 *D*; Table 1). The difference between the true stiffness and the effective stiffness was also shown to be reduced on stiffer substrates or arrangements with greater adhered area.

When considering adhesive spots (FAs) we demonstrated that both increasing the number of spots and total adhered area through increasing spot size effectively “stiffens” the substrate (Table 1; Figs. 2 and 3 *A*). Where the points of adhesion are randomly distributed, the cell shapes qualitatively look more realistic (Fig. 5, *C*–*F*) and the introduction of variation in the interspot spacing further “softens” the surface (Fig. 5 *A*).

We considered further the substrate strain energy, i.e., the work the cell does to the underlying substrate, for both adhesive ring and spots. We showed that where adhesive spots are distributed in a region around the cell edge this is energetically favorable compared with maintaining adhesions directly at the cell edge (Fig. 5 *B*), while the mean deformation is also increased, at least for the more regular arrangements (Fig. 5 *A*). We additionally show that, although FA growth would by itself reduce the mean deformation and effectively “stiffen” the substrate, the elongation of the adhesion spot can compensate for this effect (Fig. 3). On soft substrates spot elongation also reduces the substrate strain energy, $W_{s}$ (Fig. 4 *F*). However, on stiff substrates spot elongation only reduces the work done up to an optimal elongation (Fig. 4 *G*). Indeed we predict an optimal spot elongation of 2–3, for e.g., γ=15 across a range of adhesion profiles. (A resistance parameter of γ=15 corresponds to a Young’s modulus for the substrate of $E_{S}\approx 318$ kPa, with cell parameters $E_{c}=10$ kPa, radius $r_{0}=30$ *μ*m, thickness $h_{c}=1$ *μ*m, cell and substrate Poisson’s ratios $\nu_{c}=\nu_{S}=0.45$, and substrate thickness $h_{S}=35$ *μ*m (54,56).)

We note that it is observed experimentally that FAs form on stiff substrates with soft substrates having no stable adhesions, and that FAs grow and elongate on these substrates (11,16). Significantly, we here show that starting with nascent adhesions of small area, FA growth and elongation would be energetically favorable on stiff substrates but not on soft substrates (Fig. 4, *D* and *E*). We thus suggest an underpinning mechanism driving the observed FA dynamics based on energy considerations.

## Conclusion

Our results indicate that mechanotransduction studies require a consideration of the whole combined cell and substrate system moving beyond a focus on individual FAs. It is clear that there is a need for integrating theoretical modeling with experimental investigations to enable the full complexity of the system to be accounted for. However, taking this forward into mechanotransduction studies is nontrivial. Although fluorescence imaging and segmentation of FAs is a well-established technique, it is still technically complex and the adhesion patterns vary greatly even between cells on the same surface. In this context, studies in which cell adhesion is directly controlled through micropatterning techniques (17,18) could have significant advantages; where the adhesion patterns are determined a priori this can be controlled for across experiments.

## Author contributions

J.S.-W. carried out simulations and analyzed data. C.M.D., study design and data analysis. J.S.-W. and C.M.D. wrote the article. The authors declare no competing interests.

## Supporting material

Document S1. Table S1 and Figures S1–S5

Document S2. Article plus supporting material

## Footnotes

Supporting material can be found online at https://doi.org/10.1016/j.bpj.2022.03.017.
