# Supplementary material for: Sticking around: Cell adhesion patterning for energy minimization and substrate mechanosensing
Source: Biophys J. 2022 Mar 16;121(9):1777–86. doi: 10.1016/j.bpj.2022.03.017 (PMC9117892; doi:10.1016/j.bpj.2022.03.017)
Supplement: Document S1. Table S1 and Figures S1–S5 [file mmc1.pdf]

Biophysical Journal, Volume *121*

Supplemental information

Sticking around: Cell adhesion patterning for energy minimization and substrate mechanosensing

Josephine Solowiej-Wedderburn and Carina M. Dunlop

Supplementary Calculations: Optimal cell adhesion patterning for energy minimization and substrate mechanosensing

Josephine Solowiej-Wedderburn[1] and Carina Dunlop[1,2]

[1]Department of Mathematics, University of Surrey, Guildford, GU2 7XH,UK

[2]Centre for Mathematical and Computational Biology,University of Surrey, Guildford, UK

February 18, 2022

Typical values of substrate stiffness parameter γ

The key control parameter $\gamma^2 = K(1-\nu^2)r_0^2/hE$ quantifies the substrate resistance compared with that of a cell. The parameters are the Young's modulus E and Poisson ratio ν of a cell of diameter $2r_0$ and thickness h. Although these parameters can vary greatly as an indicative value we calculate that for a gel of thickness 35μm with Young's modulus $E_S = 70$kPa, $\gamma = 7$ (cell parameters $E = 10$kPa, $r_0 = 30\mu$m, $h = 1\mu$m, and $\nu = \nu_S = 0.45$). Parameters ranges and sources are listed in Table S.1.

Parameter	Value
E	mycroglia (neural cells) $\sim$ 100Pa [1]; human mesenchymal stem cells (hMSCs) $\sim$ 2–8kPa [2]; osteoblasts (bone-synthesising cells) $\sim$ 7–18kPa [3]
h	1–3μm [4]
ν	0.38–0.5 [1, 3]
r_0	22–44μm [2]
E_S	softer $\sim$ 1kPa; stiffer: 35kPa–1MPa [5]
h_S	$\sim$ 35 μm [5]
ν_S	$\sim$ 0.45 [6]

Table S.1: Parameters values used to estimate an indicative value of γ

Details of numerical implementation

Numerical solutions were only required when considering adhesion distributed in spots both for regular arrangements of spots and random distributions. The geometries considered and their definitions are listed in the main paper. For each case, a mesh was generated automatically from the defined geometry using the generateMesh command within the MATLAB PDE Toolbox (with `Hmax`, maximum edge length, set at 0.02).

To numerically solve the force balance equation (S.1) the partial differential equation is input into the PDE Toolbox in MATLAB (R2018a) as a general elliptic PDE in two dimensions

$$-\nabla \cdot (c_{11}\nabla u_1) - \nabla \cdot (c_{12}\nabla u_2) + a_{11}u_1 + a_{12}u_2 = f_1$$
$$-\nabla \cdot (c_{21}\nabla u_1) - \nabla \cdot (c_{22}\nabla u_2) + a_{21}u_1 + a_{22}u_2 = f_2,$$

where specifically the coefficient matrices are

$$c_{11} = \begin{pmatrix} 1 & 0 \\ 0 & \frac{(1-\nu)}{2} \end{pmatrix}, \; c_{12} = \begin{pmatrix} 0 & \nu \\ \frac{(1-\nu)}{2} & 0 \end{pmatrix}, \; c_{21} = \begin{pmatrix} 0 & \frac{(1-\nu)}{2} \\ \nu & 0 \end{pmatrix}, \; c_{22} = \begin{pmatrix} \frac{(1-\nu)}{2} & 0 \\ 0 & 1 \end{pmatrix},$$

$(a_{11}, a_{22}) = T(\mathbf{x})\gamma^2$, and $a_{12} = a_{21} = f_1 = f_2 = 0$. The no stress boundary condition is input as generalised Neumann boundary conditions

$$\mathbf{n} \cdot (c_{11}\nabla u_1) + \mathbf{n} \cdot (c_{12}\nabla u_2) + q_{11}u_1 + q_{12}u_2 = g_1$$
$$\mathbf{n} \cdot (c_{21}\nabla u_1) + \mathbf{n} \cdot (c_{22}\nabla u_2) + q_{21}u_1 + q_{22}u_2 = g_2,$$

with $q_{11} = q_{12} = q_{21} = q_{22} = 0$ and $(g_1, g_2) = -(1+\nu)P_0/2\mathbf{n}$. As in the case of an adhered ring, we have normalised length scales by the cell radius r_0, hence we also see that the problem can be completely parametrised by γ, P_0 and ν for a general adhesion geometry.

Derivation of analytical solution for adhered annulus

In the main paper, we express the force balance equation between the cell and substrate as

$$\nabla \cdot \sigma - KT(\mathbf{x})\mathbf{u} = \mathbf{0}. \tag{S.1}$$

For a circular cell with an adhered ring, $T(\mathbf{x})$ is given by

$$T(\mathbf{x}) = \begin{cases} 0, & |\mathbf{x}| < r_1 \\ 1, & |\mathbf{x}| \in [r_1, r_0]. \end{cases} \tag{S.2}$$

We substitute (S.2) into the force balance equation (S.1), and use the radial symmetry of the problem to express the purely radial deformations as $\mathbf{u} = u(r)\mathbf{e_r}$. Hence (S.2) becomes

$$\bar{r}^2 \frac{d^2\bar{u}}{d\bar{r}^2} + \bar{r}\frac{d\bar{u}}{d\bar{r}} - \bar{u} = 0, \quad \text{on } 0 < \bar{r} < r_1/r_0 \tag{S.3}$$

$$\& \quad \bar{r}^2 \frac{d^2\bar{u}}{d\bar{r}^2} + \bar{r}\frac{d\bar{u}}{d\bar{r}} - \left(1 + \gamma^2\bar{r}^2\right)\bar{u} = 0, \quad \text{on } r_1/r_0 \leq \bar{r} < 1, \tag{S.4}$$

where length scales have been normalised by the cell radius ($\bar{r} = r/r_0$ and $\bar{u} = u/r_0$) and γ is a dimensionless parameter such that $\gamma^2 = K(1-\nu^2)r_0^2/hE_c$.

We find the general solution to (S.3) on the inner region by making the ansatz $u \propto r^n$ to find $n = \pm 1$. This gives the general solution in $0 < \bar{r} < r_1/r_0$ that $\bar{u} = A_0\bar{r} + B_0 1/\bar{r}$. Equation (S.4) is the modified Bessel equation [7] and so the general solution on the adhered ring can be expressed in terms of modified Bessel functions as $\bar{u} = A_1 I_1(\gamma\bar{r}) + B_1 K_1(\gamma\bar{r})$. The terms A_0, B_0, A_1 and B_1 are then determined from the boundary conditions: $\bar{u}(\bar{r} = 0)$, continuity of stress and deformation at $\bar{r} = r_1/r_0$ and the zero stress condition at the outer boundary, $\sigma \cdot \mathbf{n} = 0$. This gives the solution presented in the main paper. Note that for a circular cell the condition that there is no stress at $\bar{r} = 1$ reduces to

$$\left.\frac{d\bar{u}}{d\bar{r}}\right|_{\bar{r}=1} + \nu\bar{u}(\bar{r} = 1) = \frac{-P_0(1+\nu)}{2}. \tag{S.5}$$

We see from (S.3)–(S.5) that the problem and normalised cell deformation is completely parametrised by γ, P_0 and ν, while other parameters may be used to relate γ to specific experimental scenarios.

Deriving the analytical expressions for mean cellular deformation

The mean cellular deformation is obtained from

$$\langle u \rangle = \frac{1}{A}\int_A u \, dA, \tag{S.6}$$

where A is the cell area and u the deformation. For a cell with an adhered ring the cell deformation is found in the main paper as

$$\frac{u}{P_0 r_0} = \begin{cases} \alpha_0 \frac{r}{r_0}, & r \in [0, r_1) \\ \alpha_1 I_1(\frac{\gamma r}{r_0}) + \beta_1 K_1(\frac{\gamma r}{r_0}), & r \in [r_1, r_0], \end{cases} \tag{S.7}$$

where $\alpha_0 = \frac{r_0}{r_1}\left(\alpha_1 I_1\left(\frac{\gamma r_1}{r_0}\right) + \beta_1 K_1\left(\frac{\gamma r_1}{r_0}\right)\right)$, $\alpha_1 = -\frac{(1+\nu)}{2\gamma} \cdot \left(F(\gamma) - G(\gamma) H\left(\frac{\gamma r_1}{r_0}\right)\right)^{-1}$, $\beta_1 = H\left(\frac{\gamma r_1}{r_0}\right)\alpha_1$, and

$$F(z) = I_0(z) + \frac{(\nu-1)}{z} I_1(z), \;\; G(z) = K_0(z) - \frac{(\nu-1)}{z} K_1(z), \;\; H(z) = \left(\frac{z I_0(z) - 2 I_1(z)}{z K_0(z) + 2 K_1(z)}\right).$$

Substituting this solution (S.7) into (S.6), we find

$$\begin{aligned} \langle u/r_0 \rangle &= \frac{2P_0}{r_0}\left(\int_0^{r_1} \alpha_0 \frac{r}{r_0} r dr + \int_{r_1}^{r_0} \alpha_1 I_1(\frac{\gamma r}{r_0}) r dr + \int_{r_1}^{r_0} \beta_1 K_1(\frac{\gamma r}{r_0}) r dr\right) \\ &= \frac{2P_0 \alpha_0 r_1^3}{3 r_0^3} + \frac{P_0 \alpha_1 \pi}{\gamma}\Bigg(L_0(\gamma) I_1(\gamma) - L_1(\gamma) I_0(\gamma) \\ &\qquad - \frac{r_1}{r_0}\left[L_0\left(\frac{\gamma r_1}{r_0}\right) I_1\left(\frac{\gamma r_1}{r_0}\right) - L_1\left(\frac{\gamma r_1}{r_0}\right) I_0\left(\frac{\gamma r_1}{r_0}\right)\right]\Bigg) \\ &\quad + \frac{P_0 \beta_1 \pi}{\gamma}\Bigg(L_0(\gamma) K_1(\gamma) + L_1(\gamma) K_0(\gamma) \\ &\qquad - \frac{r_1}{r_0}\left[L_0\left(\frac{\gamma r_1}{r_0}\right) K_1\left(\frac{\gamma r_1}{r_0}\right) + L_1\left(\frac{\gamma r_1}{r_0}\right) K_0\left(\frac{\gamma r_1}{r_0}\right)\right]\Bigg). \end{aligned} \tag{S.8}$$

To evaluate the final two integrals we have used the results [8] that

$$\int z I_1(z) dz = \frac{\pi}{2} z \left(L_0(z) I_1(z) - L_1(z) I_0(z)\right),$$
$$\int z K_1(z) dz = \frac{\pi}{2} z \left(L_0(z) K_1(z) + L_1(z) K_0(z)\right),$$

which are given in terms of modified Struve functions $L_0(z)$ and $L_1(z)$ (for more on these functions see [7]). Struve functions are implemented as standard functions in e.g. MATLAB, SciPy and R.

To determine the *effective substrate stiffness* γ_e for an adhesive pattern, the mean deformation is compared to that of a completely adhered cell. The deformation of a completely adhered cell is given by

$$\frac{u_{CD}}{r_0} = \frac{-P_0(1+\nu)}{2\gamma F(\gamma)} I_1\left(\frac{\gamma r}{r_0}\right). \tag{S.9}$$

Substituting (S.9) into (S.6), we determine the mean deformation of a completely adhered cell, again in terms of Struve functions, as

$$\langle u_{CD}/r_0 \rangle = \frac{P_0(1+\nu)\pi}{2\gamma^2 F(\gamma)} \left(L_1(\gamma) I_0(\gamma) - L_0(\gamma) I_1(\gamma) \right).$$

Supporting References

[1] J Rheinlaender, A Dimitracopoulos, B Wallmeyer, N M Kronenberg, K J Chalut, M C Gather, T Betz, G Charras, and K Franze. Cortical cell stiffness is independent of substrate mechanics. *Nat Mater*, 19(9):1019–1025, 2020.

[2] S-Y Tee, J Fu, C S Chen, and P A Janmey. Cell shape and substrate rigidity both regulate cell stiffness. *Biophys J*, 100(5):L25–L27, 2011.

[3] CA Mullen, TJ Vaughan, MC Voisin, MA Brennan, P Layrolle, and LM McNamara. Cell morphology and focal adhesion location alters internal cell stress. *J R Soc Interface*, 11(101):20140885, 2014.

[4] PW Oakes, S Banerjee, MC Marchetti, and ML Gardel. Geometry regulates traction stresses in adherent cells. *Biophys J*, 107(4):825–833, 2014.

[5] M Prager-Khoutorsky, A Lichtenstein, R Krishnan, K Rajendran, A Mayo, Z Kam, B Geiger, and A Bershadsky. Fibroblast polarization is a matrix-rigidity-dependent process controlled by focal adhesion mechanosensing. *Nat Cell Biol*, 13(12):1457–1465, 2011.

[6] T Takigawa, Y Morino, K Urayama, and T Masuda. Poisson's ratio of polyacrylamide (paam) gels. *Polymer Gels and Networks*, 4(1):1–5, 1996.

[7] M Abramowitz and IA Stegun. *Handbook of mathematical functions: with formulas, graphs, and mathematical tables*, volume 55. Courier Corporation, 1964.

[8] *NIST Digital Library of Mathematical Functions*. http://dlmf.nist.gov/, Release 1.1.1 of 2021-03-15. F. W. J. Olver, A. B. Olde Daalhuis, D. W. Lozier,

B. I. Schneider, R. F. Boisvert, C. W. Clark, B. R. Miller, B. V. Saunders, H. S. Cohl, and M. A. McClain, eds.

Supplementary Information: Figures

S1 Fig. On stiffer substrates, cells require a thinner ring to achieve the same proportion of effectively experienced resistance. Heat map shows relative effective resistance plotted against internal ring radius (r_1/r_0) and γ; $\nu = 0.45$ and $P_0 = 0.7$.

S2 Fig. Other quantitative measures demonstrate similar effects to mean cellular deformation. Heat maps show (A) the maximum cellular deformation, (B) maximum cellular strain, and (C) mean cellular strain, plotted against ring thickness (parameterised by r_1) and substrate stiffness (parameterised by γ). For each postulated measure, we would predict the same effective resistance experienced along the contour lines (black) which show a constant value of the given measure. These appear qualitatively very similar across the plots.

S3 Fig. $\mathbf{W_{CA}}$ dynamics are qualitatively very similar to the mean cellular deformation. Active work done by the cell plotted against adhered area for cells with different numbers of evenly distributed adhesive spots; $\gamma = 7$, $\nu = 0.45$, $P_0 = 0.7$.

S4 Fig. Focal Adhesion growth reduces strain energy on stiff substrates. Heat map shows substrate strain energy resulting from a cell with an adhered ring against inner ring radius (r_1/r_0) and substrate stiffness (γ).

S5 Fig. Random placement of adhesion sites for stiff substrates with $\gamma = 15$. (A) Mean cellular deformation, and (B) substrate strain energy, plotted against the variance in angular gap size of adjacent spots from 20 simulations of spots around a cell edge and corresponding ‘ring’ distributions with the same angular placements but radial positions distributed within an annular region

between $0.6r_0$ and the cell edge. Spot distributions are identical to those in Figs 5A and B. Results for an even distribution of spots are included for comparison.

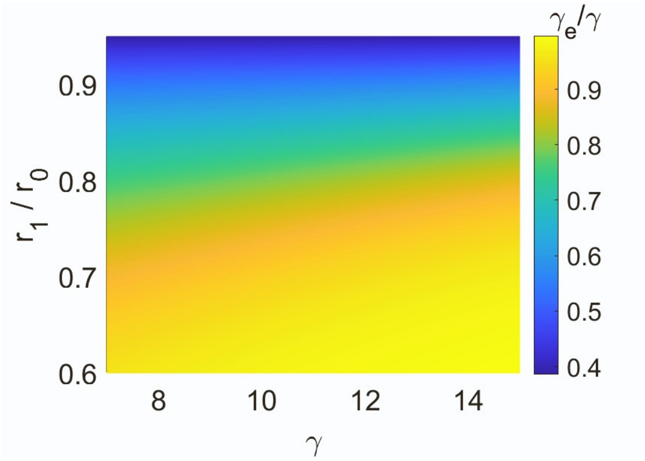

Figure S.1: **On stiffer substrates, cells require a thinner ring to achieve the same proportion of effectively experienced resistance.** Heat map shows relative effective resistance plotted against internal ring radius (r_1/r_0) and γ; $\nu = 0.45$ and $P_0 = 0.7$.

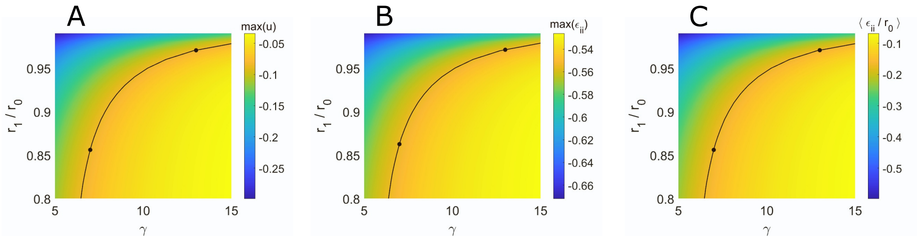

Figure S.2: **Other quantitative measures demonstrate similar effects to mean cellular deformation.** Heat maps show (A) the maximum cellular deformation, (B) maximum cellular strain, and (C) mean cellular strain, plotted against ring thickness (parameterised by r_1) and substrate stiffness (parameterised by γ). For each postulated measure, we would predict the same effective resistance experienced along the contour lines (black) which show a constant value of the given measure. These appear qualitatively very similar across the plots.

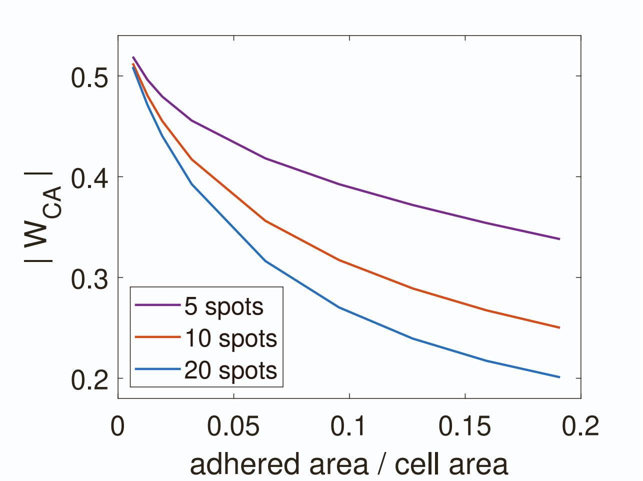

Figure S.3: **$\mathbf{W_{CA}}$ dynamics are qualitatively very similar to the mean cellular deformation.** Active work done by the cell plotted against adhered area for cells with different numbers of evenly distributed adhesive spots; $\gamma = 7$, $\nu = 0.45$, $P_0 = 0.7$.

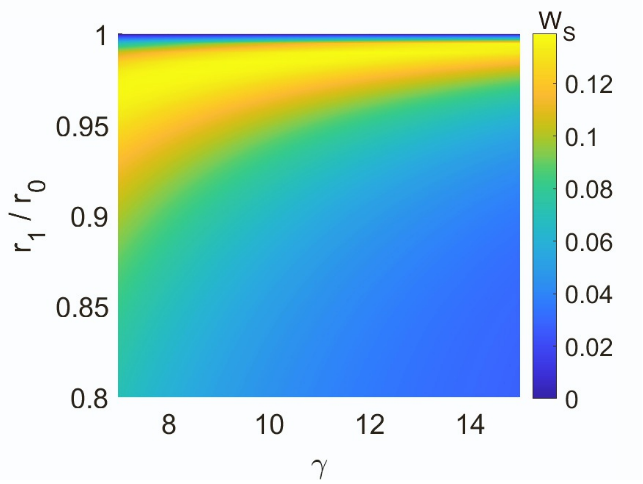

Figure S.4: **Focal Adhesion growth reduces strain energy on stiff substrates.** Heat map shows substrate strain energy resulting from a cell with an adhered ring against inner ring radius (r_1/r_0) and substrate stiffness (γ).

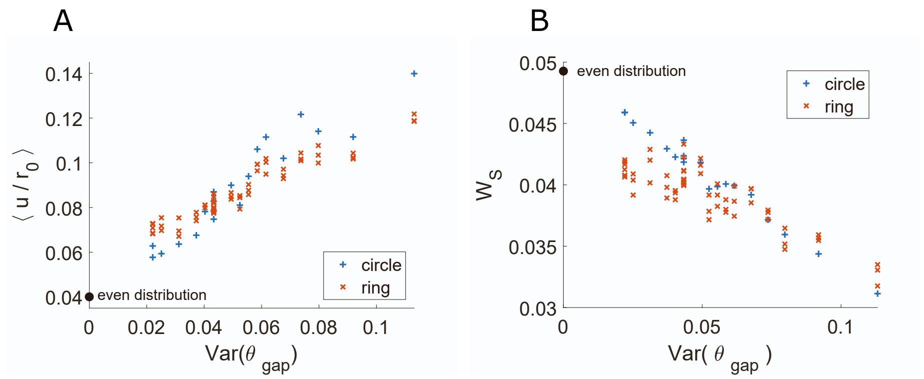

Figure S.5: **Random placement of adhesion sites for stiff substrates with $\gamma = 15$.** (A) Mean cellular deformation, and (B) substrate strain energy, plotted against the variance in angular gap size of adjacent spots from 20 simulations of spots around a cell edge and corresponding 'ring' distributions with the same angular placements but radial positions distributed within an annular region between $0.6r_0$ and the cell edge. Spot distributions are identical to those in Figs 5A and B. Results for an even distribution of spots are included for comparison.
